# Molecular Surveillance Identifies Multiple Transmissions of Typhoid in West Africa

**DOI:** 10.1371/journal.pntd.0004781

**Published:** 2016-09-22

**Authors:** International Typhoid Consortium, Vanessa K. Wong, Kathryn E. Holt, Chinyere Okoro, Stephen Baker, Derek J. Pickard, Florian Marks, Andrew J. Page, Grace Olanipekun, Huda Munir, Roxanne Alter, Paul D. Fey, Nicholas A. Feasey, Francois-Xavier Weill, Simon Le Hello, Peter J. Hart, Samuel Kariuki, Robert F. Breiman, Melita A. Gordon, Robert S. Heyderman, Jan Jacobs, Octavie Lunguya, Chisomo Msefula, Calman A. MacLennan, Karen H. Keddy, Anthony M. Smith, Robert S. Onsare, Elizabeth De Pinna, Satheesh Nair, Ben Amos, Gordon Dougan, Stephen Obaro

**Affiliations:** 1 The Wellcome Trust Sanger Institute, Hinxton, Cambridge, United Kingdom; 2 Addenbrooke’s Hospital, Cambridge University Hospitals National Health Service Foundation Trust, Cambridge, United Kingdom; 3 Department of Biochemistry and Molecular Biology, Bio21 Molecular Science and Biotechnology Institute, University of Melbourne, Parkville, Victoria, Australia; 4 Centre for Systems Genomics, University of Melbourne, Parkville, Victoria, Australia; 5 The Hospital for Tropical Diseases, Wellcome Trust Major Overseas Programme, Oxford University Clinical Research Unit, Ho Chi Minh City, Vietnam; 6 Centre for Tropical Medicine and Global Health, Nuffield Department of Clinical Medicine, Oxford University, Oxford, United Kingdom; 7 Department of Infectious and Tropical Diseases, London School of Hygiene and Tropical Medicine, London, United Kingdom; 8 International Vaccine Institute, Department of Epidemiology, Kwanak, Seoul, Republic of Korea; 9 International Foundation Against Infectious Diseases in Nigeria, Abuja, Nigeria; 10 Department of Medical Microbiology, Aminu Kano Teaching Hospital, Kano, Nigeria; 11 Department of Pathology and Microbiology, University of Nebraska Medical Center, Omaha, Nebraska, United States of America; 12 Liverpool School of Tropical Medicine, Pembroke Place, Liverpool, United Kingdom; 13 Institut Pasteur, Unité des Bactéries Pathogènes Entériques, Paris, France; 14 Institute of Biomedical Research, School of Immunity and Infection, College of Medicine and Dental Sciences, University of Birmingham, Birmingham, United Kingdom; 15 St George’s University of London, London, United Kingdom; 16 Kenya Medical Research Institute (KEMRI), Nairobi, Kenya; 17 Centers for Disease Control and Prevention, Atlanta, Georgia, United States of America; 18 Emory Global Health Institute, Atlanta, Georgia, United States of America; 19 Institute of Infection and Global Health, University of Liverpool, United Kingdom; 20 Malawi-Liverpool-Wellcome-Trust Clinical Research Programme, College of Medicine, University of Malawi, Chichiri, Blantyre, Malawi; 21 Division of Infection and Immunity, University College London, London, United Kingdom; 22 Department of Clinical Sciences, Institute of Tropical Medicine, Antwerp, Belgium; 23 KU Leuven, University of Leuven, Department of Microbiology and Immunology, Belgium; 24 National Institute for Biomedical Research, Kinshasa, Democratic Republic of the Congo; 25 University Hospital of Kinshasa, Kinshasa, Democratic Republic of the Congo; 26 Microbiology Department, College of Medicine, University of Malawi, Malawi; 27 The Jenner Institute, Nuffield Department of Medicine, University of Oxford, Oxford, United Kingdom; 28 Centre for Enteric Diseases, National Institute for Communicable Diseases, Division in the National Health Laboratory Service and Faculty of Health Sciences, University of the Witwatersrand, Johannesburg, South Africa; 29 Salmonella Reference Service, Public Health England, Colindale, London, United Kingdom; 30 St Augustine’s Hospital, Muheza, Tanzania; 31 Division of Pediatric Infectious Diseases, University of Nebraska Medical Center, Omaha, Nebraska, United States of America; 32 University of Abuja Teaching Hospital, Gwagwalada, Nigeria; 33 Bingham University, Karu, Nassarawa State, Nigeria; Massachusetts General Hospital, UNITED STATES

## Abstract

**Background:**

The burden of typhoid in sub-Saharan African (SSA) countries has been difficult to estimate, in part, due to suboptimal laboratory diagnostics. However, surveillance blood cultures at two sites in Nigeria have identified typhoid associated with *Salmonella enterica* serovar Typhi (*S*. Typhi) as an important cause of bacteremia in children.

**Methods:**

A total of 128 *S*. Typhi isolates from these studies in Nigeria were whole-genome sequenced, and the resulting data was used to place these Nigerian isolates into a worldwide context based on their phylogeny and carriage of molecular determinants of antibiotic resistance.

**Results:**

Several distinct *S*. Typhi genotypes were identified in Nigeria that were related to other clusters of *S*. Typhi isolates from north, west and central regions of Africa. The rapidly expanding *S*. Typhi clade 4.3.1 (H58) previously associated with multiple antimicrobial resistances in Asia and in east, central and southern Africa, was not detected in this study. However, antimicrobial resistance was common amongst the Nigerian isolates and was associated with several plasmids, including the IncHI1 plasmid commonly associated with *S*. Typhi.

**Conclusions:**

These data indicate that typhoid in Nigeria was established through multiple independent introductions into the country, with evidence of regional spread. MDR typhoid appears to be evolving independently of the haplotype H58 found in other typhoid endemic countries. This study highlights an urgent need for routine surveillance to monitor the epidemiology of typhoid and evolution of antimicrobial resistance within the bacterial population as a means to facilitate public health interventions to reduce the substantial morbidity and mortality of typhoid.

## Author Summary

Typhoid fever, a serious bloodstream infection caused by the bacterium *Salmonella* Typhi, is a major cause of disease and death around the world. There have been limited data on the epidemiology of typhoid in many countries in sub-Saharan African, including Nigeria. Recent evidence, however, showed that typhoid was an important cause of bacteraemia in children residing in two regions of Nigeria. Here, we analyzed the whole genome sequences of 128 *S*. Typhi isolates from two studies in order to elucidate the population structure and characterize the genetic components of antimicrobial resistance. We found that the multiple *S*. Typhi genotypes identified were closely related to other *S*. Typhi from neighboring regions of Africa and that multidrug resistance (MDR) was common among these isolates, and in many cases was associated with the IncHI1 plasmid known to cause MDR typhoid. These results provide evidence that typhoid was established in Nigeria as a result of several independent introductions into the country and that there has been extensive exchange of *S*. Typhi in and around the region of West Africa. This study emphasizes the importance of surveillance to improve our understanding of the epidemiology of typhoid, which is needed to underpin public health measures to reduce the spread of disease and facilitate patient management.

## Introduction

Typhoid fever is a systemic infection caused by the Gram-negative bacterium *Salmonella enterica* serovar Typhi (*S*. Typhi) that continues to be a serious global health problem and a major cause of morbidity and mortality in low-middle income countries [1]. It is estimated that the yearly incidence of typhoid fever exceeds 20 million cases, with over 200,000 deaths [2, 3]. Defining the burden of typhoid fever is a challenge in settings where there are few diagnostic microbiology facilities, with diagnosis often based on clinical history of fever, malaise, and abdominal pain. Unfortunately, these symptoms have considerable overlap with several other febrile illnesses and clinical diagnosis is therefore inaccurate [4].

Nigeria is one of the most densely populated countries in Africa with large areas of urban development. Thus, it is perhaps surprising that little reliable data are available on microbial culture of the etiologic agents of bacteremia in children or adults. This poses a challenge for data comparison with other regions, including other sub-Saharan African countries where such data are available [5–7]. In general, febrile illnesses among children in Nigeria are presumed by clinicians to be caused by malaria, which is still very common in many parts of the country. Only if fever persists following an empiric course of anti-malarials, is typhoid then considered as a potential cause of infection [8]. In studies from central and northwest Nigeria [9], we found that *S*. Typhi was the commonest cause of bloodstream infections in children, particularly in those living in the proximity of Abuja city located in central Nigeria.

Until recently, molecular epidemiological studies on *S*. Typhi were compromised by a lack of genetic resolution, limiting the ability to define the population structure of the bacteria and identify transmission patterns. This is because *S*. Typhi is a relatively monomorphic pathogen with limited genome variation [10]. However, sequencing-based approaches have facilitated the stratification of *S*. Typhi into multiple genotypes [11] (see Wong *et al*. 2016, under review in *Nature Communications*, NCOMMS-15-25823, manuscript included). Whole genome sequencing in particular can unequivocally identify phylogenetic relationships with important genetic traits such as antimicrobial resistance [12]. Here we report whole genome-based analysis of 128 bloodstream isolates of *S*. Typhi from children residing in two regions of Nigeria, and compared these with data from other countries in Africa, including the West African subregion.

## Methods

### Settings

Nigeria has a population of approximately 177 million people making it the most populous country in sub-Saharan Africa [13]. The two study sites in Nigeria were the Federal Capital Territory (FCT) and Kano. The FCT is a federal territory in central Nigeria and covers a land area of 8,000 square kilometers. It is the home of the capital city Abuja, a “planned” city, built in the 1980s. It was officially made Nigeria’s capital in 1991 replacing the previous capital in Lagos. In 2006, the population was estimated at 1.7 million [14]. The FCT continue to experience rapid population growth; it has been reported that some areas around Abuja have been growing at an annual rate of 20–30%, and the current population may be as high as 5.7 million [14]. The rapid spread of squatter settlements and shantytowns in and around the city limits contribute to this rapid growth. The rainy season begins in April and ends in October. Within this period there is a brief interlude of Harmattan, occasioned by the Northeast Trade Wind, with the main features of dust haze, intensified coldness and dryness. The annual total rainfall for the FCT is in the range of 1,100 to 1,600 mm. The population is diverse, with increasing representation from the major ethnic groups of Hausa, Yoruba, and Igbos following the development of the FCT and relocation of the federal capital [15]. Of note, there is also perennial malaria transmission, mostly due to *Plasmodium falciparum*, and the HIV prevalence is 7.5% amongst pregnant women attending antenatal clinics [16].

Kano is the capital of Kano state in northwest Nigeria. According to the 2006 census, Kano state has a population of 9.38 million, which is comprised predominantly of Hausa and Fulani ethnic groups [17]. It is recognized as one of the fastest growing cities in Nigeria with a population density of about 1,000 inhabitants per km^2^. It lies within the Sahel savannah region with daily mean temperature of about 30–33°C during the dry months of March to May and 10°C during the autumn months of September to February. Rainy season varies from year to year, but typically commences in May and ends in October, with an average annual rainfall of 600mm. The dry season starts from November to April [18]. The entire state is within the meningococcal disease belt and malarial transmission is seasonal [17]. HIV prevalence among women attending antenatal clinic is 1.3% [16].

### Enrolment sites

The enrolment sites at FCT are as previously described [9, 15]. Briefly, children aged less than 5 years were enrolled from primary, secondary and tertiary healthcare facilities on presentation with an acute febrile illness and symptoms suggestive of sepsis. In Kano, we enrolled children from Aminu Kano Teaching Hospital (AKTH), Hasiya Bayero Pediatric Hospital and Murtala Specialist Hospital. While AKTH serves as a tertiary referral center, the other two facilities provide primary and secondary healthcare services. The combined outpatient attendance for children at these three facilities is about 1,000 daily. Both study sites included patients from the newer settlements on the outskirt of Abuja and around Kano where the level of sanitation is poor and access to potable water limited.

### Data collection

A structured questionnaire was used to collate the clinical information. Study data were collected and managed using REDCap electronic data capture tools hosted at the University of Nebraska Medical Center [19]. IBM SPSS for statistics was used for data analysis. Dichotomous variables were analyzed using *χ*^*2*^ or *χ*^*2*^ for trend tests [20].

### Ethics statement

Clinical information was collected using a structured questionnaire after obtaining a signed informed consent from the child’s parent or legal guardian. This study was approved by the ethics committees of the FCT, National Hospital Abuja, Zankli Medical Center, Federal Medical Center Keffi, Aminu Kano Teaching Hospital, and UNMC, Omaha Institutional Review Board.

### Blood culture processing

Blood sampling and processing were as previously described [9, 15]. Briefly, we utilized only aerobic blood culture bottles and held cultures in the Bactec 9050 incubator for a maximum of 5 days. Bacteria were identified by a combination of colony morphology and biochemical assays. For example, the API 20E system (bioMérieux, France) was used to identify *Enterobacteriacae*. Antimicrobial susceptibility profiles of the bacteria were determined by the Kirby-Bauer disk diffusion test using standard interpretative criteria [21] for locally available antimicrobials (amoxicillin, co-amoxiclav, ceftazidime, ceftriaxone, nalidixic acid, ciprofloxacin, ofloxacin, sulfamethoxazole, trimethoprim-sulfamethoxazole, chloramphenicol, tetracycline, streptomycin, gentamicin, kanamycin, azithromycin, imipenem) in order to provide immediate management of patients. Bacterial isolates were stored in skimmed milk at -70°C and further characterized at the Clinical Microbiology Laboratory of the University of Nebraska Medical Center (UNMC).

### Antimicrobial susceptibility testing

Antimicrobial susceptibility testing was performed at the UNMC Microbiology laboratory using the Epsilometer test (Etest; bioMérieux, France) according to standard methods. Minimum inhibitory concentration (MIC) values were interpreted according to Clinical Laboratory Standards Institute (CLSI) standards [21]. Due to the lack of CLSI standards, a streptomycin MIC of ≥16 mg/L was considered resistant in these studies.

### *Salmonella* serotyping

All *Salmonella* isolates were identified to the serotype level using the Bioplex 200 (Bio-Rad) as previously described using the CDC standard *Salmonella* molecular serotyping protocol [22–24]. A total of 128 *S*. Typhi isolates were identified in these studies for whole genome sequencing.

### DNA sequencing

*S*. Typhi DNA was prepared using the Wizard Genomic DNA Kit (Promega, Madison, WI, USA) as per manufacturer’s instructions. Index-tagged paired end Illumina sequencing libraries were prepared as previously described [25]. These were combined into pools each containing 96 uniquely tagged libraries and sequenced on the Illumina Hiseq2000 or Miseq platforms (Illumina, San Diego, CA, USA) according to manufacturer’s protocols to generate tagged 100 or 150 base pair (bp) paired-end reads with an insert size of 300–400 bp. Sequence reads were deposited in the European Nucleotide Archive under accession ERP005877 and a full list of accession numbers for each sample is available in S1 Table. Sequence data from 1,831 additional *S*. Typhi isolates from 63 countries, generated previously in the same manner (Wong *et al*. 2015) [12], were also included in the study (reads are available in the European Read Archive under accession ERP001718).

### Read alignment and SNP detection

For analysis of single nucleotide polymorphisms (SNPs), the paired-end reads were mapped to the reference genome of *S*. Typhi CT18 (accession number AL513382), including the chromosome and plasmids pHCM1 and pHCM2 [26], using SMALT (version 0.7.4) (http://www.sanger.ac.uk/resources/software/smalt/). SNPs were identified as previously described, using *samtools mpileup* [27] and filtering with a minimum mapping quality of 30 and a quality ratio cut-off of 0.75 [25]. The allele at each locus in each isolate was determined by reference to the consensus base in that genome, using *samtools mpileup* [27] and removing low confidence alleles with consensus base quality ≤20, read depth ≤5 or a heterozygous base call. SNPs called in phage regions, repetitive sequences (354 kbp; ~7.4% of bases in the *S*. Typhi CT18 reference chromosome, as defined previously [10]) or recombinant regions (~180 kbp; <4% of CT18 reference chromosome, identified using an approach described previously [25, 28]) were excluded, resulting in a final set of 23,300 chromosomal SNPs.

### Phylogenetic analysis

The maximum likelihood (ML) phylogenetic tree was built from 23,300 SNP alignment of 1,961 isolates, including one *S*. Paratyphi A (accession number ERR326600) to provide an outgroup for tree rooting. We used RAxML (version 7.0.4) [29] with the generalized time-reversible model and a Gamma distribution to model site-specific rate variation (the GTR+ substitution model; GTRGAMMA in RAxML). Support for the ML phylogeny was assessed via 100 bootstrap pseudo-replicate analyzes of the alignment data. The ML trees were displayed and annotated using iTOL [30, 31].

### *In silico* resistance plasmid and resistance gene analysis

Plasmids and acquired antimicrobial resistance genes were detected, and their precise alleles determined, using the mapping-based allele typer SRST2 [32] together with the ARG-Annot database of antimicrobial resistance genes [33] and the PlasmidFinder database of plasmid replicons [34]. SRST2 was also used to identify mutations in the *gyrA*, *gyrB*, *parC* and *parE* genes that have been associated with resistance to quinolones in *Salmonella* and other Gram-negative bacteria [35–38].

## Results

### Typhoid surveillance

Blood cultures were performed for the evaluation of 10,133 acutely ill children, aged 0–60 months, from September 2008 until April 2015, in the FCT (including Abuja) and Kano located in central and northwest Nigeria, respectively [9]. At FCT 6,082 children were enrolled between June 2012 and March 2015, of whom 457 (8%) had clinically significant bacteremia. Of these 110 (24%) had invasive salmonellosis, consisting of *S*. Typhi in 84 cases and non-typhoidal salmonellae (NTS) in 26 cases. In Kano from January 2014 until April 2015 clinically significant bacteremia was detected in 609 (15%) of 4,051 children: salmonellae accounted for 364 (60%) of 609 cases, of which 296 were *S*. Typhi and 68 were NTS. Across both regions *Salmonella* species accounted for 24–60% of bacteremia with *S*. Typhi being the most common serovar isolated with a total of 380 isolates (76–79%) [9].

### Phylogenetic analysis of Nigerian *S*. Typhi

**Fig 1:**
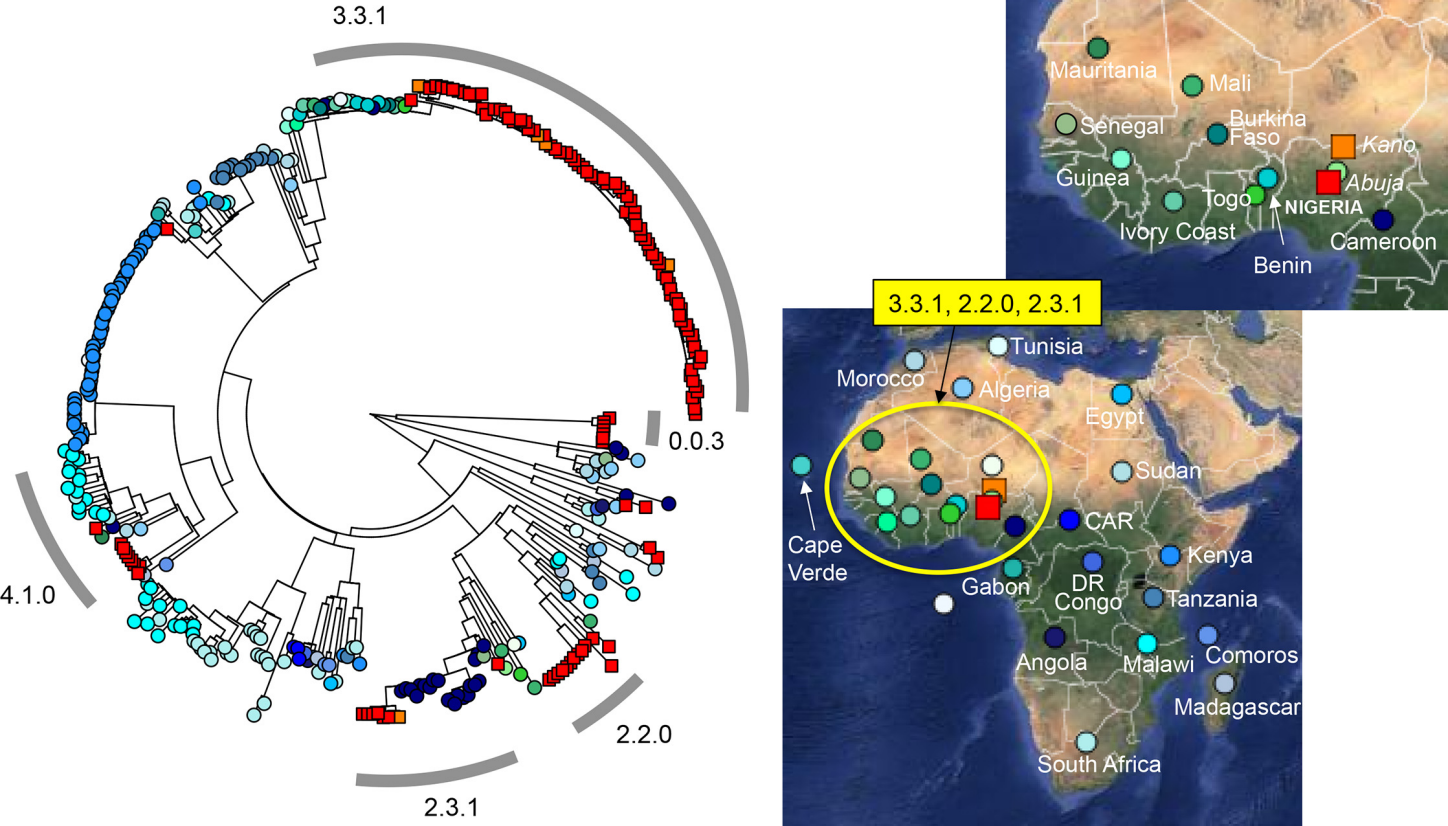
Distribution of Nigerian *S*. Typhi isolates in Africa in this study. A maximum likelihood tree of 366 *S*. Typhi isolates constructed using 9,352 SNPs from whole genome sequence from 128 Nigerian isolates and 238 isolates from other regions of Africa is shown on the left. The geographical location of isolation is highlighted on the maps of Africa displayed on the right (http://microreact.org/showcase/). *S*. Typhi isolates from Abuja (122 isolates) and Kano (6) are denoted using red and orange squares, respectively. Colored circles on both the tree and maps represent isolates from other regions of Africa. The common genotypes of the Nigerian isolate are highlighted by a grey ring surrounding the tree with the corresponding geographical location marked on the map. Branch lengths are indicative of the estimated substitution rate per variable site.

A selection of one hundred and twenty-two *S*. Typhi from the FCT and six from Kano, all isolated between 2008–2013, were randomly selected and sequenced via Illumina HiSeq and MiSeq (see Methods). The genomes of the Nigerian isolates were compared to that of the *S*. Typhi CT18 reference strain and a previously published global collection of approximately 2,000 *S*. Typhi isolates [12]. A phylogeny was built by extracting single nucleotide polymorphisms (SNPs) from the whole genome sequences, excluding likely recombination events and repetitive sequences that could confound phylogenetic analysis as described in Methods. The SNP data were also used to assign each isolate to one of 62 previously defined genotypes; details of the source and genotype of all Nigerian isolates is given in Table 1 and S1 Table. The distribution of the 128 Nigerian *S*. Typhi within the global phylogenetic tree is shown in S1 Fig. This global phylogeny includes 238 isolates from other countries in Africa, and the Nigerian isolates all cluster with other African isolates. Detailed phylogenetic relationships amongst the 366 African isolates are shown in Fig 1, and an interactive version of the phylogeny and map are available for exploration online at http://microreact.org/project/styphi_nigeria.

**Table 1:** Summary of genotypes of Nigerian *S*. Typhi.

Laboratory name	Year of isolation	Location	Roumagnac haplotype*	Primary clade	Clade	Subclade
PO_30	2008	Abuja	H56	3	3.1	3.1.1
PO_601	2009	Abuja	Untypeable	1	1	0.0.3
PO_132	2009	Abuja	Untypeable	2	2.3	2.3.1
PO_107	2009	Abuja	Untypeable	2	2.3	2.3.1
PO_1057	2009	Abuja	H56	3	3.1	3.1.1
PO_1060	2009	Abuja	H56	3	3.1	3.1.1
PO_1063	2009	Abuja	H56	3	3.1	3.1.1
PO_187	2009	Abuja	H56	3	3.1	3.1.1
PO_227	2009	Abuja	H56	3	3.1	3.1.1
PO_293	2009	Abuja	H56	3	3.1	3.1.1
PO_351	2009	Abuja	H56	3	3.1	3.1.1
PO_355	2009	Abuja	H56	3	3.1	3.1.1
PO_771	2009	Abuja	H56	3	3.1	3.1.1
PO_812	2009	Abuja	H56	3	3.1	3.1.1
PO_919	2009	Abuja	H56	3	3.1	3.1.1
PO_575	2009	Abuja	H56	3	3.1	3.1.1
PO_260	2009	Abuja	H52	4	4.1	4.1.0
PO_1102	2010	Abuja	Untypeable	1	1	0.0.3
PO_1131	2010	Abuja	Untypeable	1	1	0.0.3
3135STDY5861198	2010	Abuja	Untypeable	1	1	0.0.3
3135STDY5861206	2010	Abuja	Untypeable	1	1	0.0.3
3135STDY5861239	2010	Abuja	Untypeable	1	1	0.0.3
3135STDY5861254	2010	Abuja	Untypeable	2	2.1	2.1.0
PO_1255	2010	Abuja	H56	3	3.1	3.1.1
PO_1098	2010	Abuja	H56	3	3.1	3.1.1
PO_1101	2010	Abuja	H56	3	3.1	3.1.1
PO_1210	2010	Abuja	H56	3	3.1	3.1.1
PO_1242	2010	Abuja	H56	3	3.1	3.1.1
PO_1265	2010	Abuja	H56	3	3.1	3.1.1
3135STDY5861190	2010	Abuja	H56	3	3.1	3.1.1
3135STDY5861230	2010	Abuja	H56	3	3.1	3.1.1
3135STDY5861262	2010	Abuja	H56	3	3.1	3.1.1
3135STDY5861271	2010	Abuja	H56	3	3.1	3.1.1
3135STDY5861184	2010	Abuja	H56	3	3.1	3.1.1
3135STDY5861208	2010	Abuja	H56	3	3.1	3.1.1
3135STDY5861216	2010	Abuja	H56	3	3.1	3.1.1
3135STDY5861224	2010	Abuja	H56	3	3.1	3.1.1
3135STDY5861232	2010	Abuja	H56	3	3.1	3.1.1
3135STDY5861183	2010	Abuja	H42	3	3.3	3.3.0
PO_1232	2010	Abuja	H52	4	4.1	4.1.0
3135STDY5861222	2010	Abuja	H52	4	4.1	4.1.0
3135STDY5861246	2010	Abuja	H52	4	4.1	4.1.0
3135STDY5861270	2010	Abuja	H52	4	4.1	4.1.0
3135STDY5861199	2010	Abuja	H52	4	4.1	4.1.0
3135STDY5861247	2010	Abuja	H52	4	4.1	4.1.0
PO_1110	2010	Abuja	H52	4	4.1	4.1.1
PO_1256	2011	Abuja	Untypeable	1	1	0.0.3
3135STDY5861272	2011	Abuja	Untypeable	2	2.1	2.1.0
3135STDY5861209	2011	Abuja	Untypeable	2	2.2	2.2.0
3135STDY5861248	2011	Abuja	H56	3	3.1	3.1.1
3135STDY5861256	2011	Abuja	H56	3	3.1	3.1.1
3135STDY5861264	2011	Abuja	H56	3	3.1	3.1.1
3135STDY5861193	2011	Abuja	H56	3	3.1	3.1.1
3135STDY5861201	2011	Abuja	H56	3	3.1	3.1.1
3135STDY5861233	2012	Abuja	Untypeable	2	2.3	2.3.1
3135STDY5861241	2012	Abuja	Untypeable	2	2.3	2.3.1
3135STDY5861273	2012	Abuja	Untypeable	2	2.3	2.3.1
3135STDY5861217	2012	Abuja	H56	3	3.1	3.1.1
3135STDY5861225	2012	Abuja	H56	3	3.1	3.1.1
3135STDY5861195	2012	Abuja	Untypeable	2	2.3	2.3.1
3135STDY5861211	2012	Abuja	H56	3	3.1	3.1.1
3135STDY5861235	2012	Abuja	H56	3	3.1	3.1.1
3135STDY5861243	2012	Abuja	H56	3	3.1	3.1.1
3135STDY5861210	2013	Kano	Untypeable	2	2.3	2.3.1
3135STDY5861226	2013	Kano	H56	3	3.1	3.1.1
3135STDY5861202	2013	Kano	H56	3	3.1	3.1.1
3135STDY5861234	2013	Kano	H56	3	3.1	3.1.1
3135STDY5861242	2013	Kano	H56	3	3.1	3.1.1
3135STDY5861218	2013	Kano	H56	3	3.1	3.1.1
3135STDY5861196	2013	Abuja	Untypeable	1	1	0.0.1
3135STDY5861244	2013	Abuja	Untypeable	1	1	0.0.1
3135STDY5861359	2013	Abuja	Untypeable	2	2.2	2.2.0
3135STDY5861290	2013	Abuja	Untypeable	2	2.2	2.2.0
3135STDY5861298	2013	Abuja	Untypeable	2	2.2	2.2.0
3135STDY5861350	2013	Abuja	Untypeable	2	2.2	2.2.0
3135STDY5861287	2013	Abuja	Untypeable	2	2.2	2.2.0
3135STDY5861280	2013	Abuja	Untypeable	2	2.2	2.2.0
3135STDY5861289	2013	Abuja	Untypeable	2	2.2	2.2.0
3135STDY5861337	2013	Abuja	Untypeable	2	2.2	2.2.0
3135STDY5861353	2013	Abuja	Untypeable	2	2.2	2.2.0
3135STDY5861361	2013	Abuja	Untypeable	2	2.2	2.2.0
3135STDY5861369	2013	Abuja	Untypeable	2	2.2	2.2.0
3135STDY5861253	2013	Abuja	Untypeable	2	2.2	2.2.0
3135STDY5861342	2013	Abuja	Untypeable	2	2.3	2.3.1
3135STDY5861334	2013	Abuja	Untypeable	2	2.3	2.3.2
3135STDY5861294	2013	Abuja	H56	3	3.1	3.1.0
3135STDY5861312	2013	Abuja	H56	3	3.1	3.1.1
3135STDY5861320	2013	Abuja	H56	3	3.1	3.1.1
3135STDY5861268	2013	Abuja	H56	3	3.1	3.1.1
3135STDY5861276	2013	Abuja	H56	3	3.1	3.1.1
3135STDY5861229	2013	Abuja	H56	3	3.1	3.1.1
3135STDY5861237	2013	Abuja	H56	3	3.1	3.1.1
3135STDY5861245	2013	Abuja	H56	3	3.1	3.1.1
3135STDY5861314	2013	Abuja	H56	3	3.1	3.1.1
3135STDY5861330	2013	Abuja	H56	3	3.1	3.1.1
3135STDY5861197	2013	Abuja	H56	3	3.1	3.1.1
3135STDY5861338	2013	Abuja	H56	3	3.1	3.1.1
3135STDY5861351	2013	Abuja	H56	3	3.1	3.1.1
3135STDY5861282	2013	Abuja	H56	3	3.1	3.1.1
3135STDY5861306	2013	Abuja	H56	3	3.1	3.1.1
3135STDY5861322	2013	Abuja	H56	3	3.1	3.1.1
3135STDY5861326	2013	Abuja	H56	3	3.1	3.1.1
3135STDY5861366	2013	Abuja	H56	3	3.1	3.1.1
3135STDY5861279	2013	Abuja	H56	3	3.1	3.1.1
3135STDY5861303	2013	Abuja	H56	3	3.1	3.1.1
3135STDY5861319	2013	Abuja	H56	3	3.1	3.1.1
3135STDY5861327	2013	Abuja	H56	3	3.1	3.1.1
3135STDY5861335	2013	Abuja	H56	3	3.1	3.1.1
3135STDY5861343	2013	Abuja	H56	3	3.1	3.1.1
3135STDY5861367	2013	Abuja	H56	3	3.1	3.1.1
3135STDY5861304	2013	Abuja	H56	3	3.1	3.1.1
3135STDY5861328	2013	Abuja	H56	3	3.1	3.1.1
3135STDY5861336	2013	Abuja	H56	3	3.1	3.1.1
3135STDY5861344	2013	Abuja	H56	3	3.1	3.1.1
3135STDY5861260	2013	Abuja	H56	3	3.1	3.1.1
3135STDY5861189	2013	Abuja	H56	3	3.1	3.1.1
3135STDY5861213	2013	Abuja	H56	3	3.1	3.1.1
3135STDY5861278	2013	Abuja	H56	3	3.1	3.1.1
3135STDY5861286	2013	Abuja	H56	3	3.1	3.1.1
3135STDY5861251	2013	Abuja	H56	3	3.1	3.1.1
3135STDY5861259	2013	Abuja	H56	3	3.1	3.1.1
3135STDY5861212	2013	Abuja	H56	3	3.1	3.1.1
3135STDY5861220	2013	Abuja	H56	3	3.1	3.1.1
3135STDY5861228	2013	Abuja	H56	3	3.1	3.1.1
3135STDY5861252	2013	Abuja	H56	3	3.1	3.1.1
3135STDY5861236	2013	Abuja	H56	3	3.1	3.1.1
3135STDY5861368	2013	Abuja	Untypeable	3	3.1	3.1.1
3135STDY5861205	2013	Abuja	H52	4	4.1	4.1.0

* Reference [11]

The majority of Nigerian *S*. Typhi (84/128, 66%) belonged to genotype 3.1.1 (these isolates were assigned to H56 under the old typing scheme of Roumagnac *et al* (2006) [11]). This dominant genotype is relatively common across Africa, predominantly western and central countries (Fig 1). The Nigerian isolates formed a tight phylogenetically clustered subgroup within the 3.1.1 subclade (Fig 1), suggesting recent local expansion, and included isolates from both Abuja and Kano, suggesting intra-country transmission. Interestingly, in the wider African collection genotype 3.1.1 was represented by isolates from neighboring Cameroon and across West Africa (Benin, Togo, Ivory Coast, Burkina Faso, Mali, Guinea and Mauritania) suggesting long-term inter-country exchange within the region (Fig 1). Most of the remaining isolates belonged to four other genotypes, indicating that these are also established genotypes in circulation at the study sites in Nigeria. These genotypes, highlighted in Fig 1, are 2.2.0 (n = 13), 2.3.1 (n = 8), 4.1.0 (n = 8, H52 under the old scheme) and 0.0.3 (n = 7, H12). Nigerian isolates of genotypes 2.2.0 and 2.3.1 were closely related to isolates from neighboring Cameroon and West African countries and not found elsewhere, supporting regional transmission similar to the dominant genotype 3.1.1 (see map in Fig 1), while genotype 4.1.0 was more widespread across Africa. Interestingly genotype 0.0.3 (previously identified in India and Malaysia), which accounted for >5% of Nigerian isolates, maps very close to the root of the global *S*. Typhi tree, suggestive of older circulating isolates. A further six other genotypes were also detected amongst the Nigerian isolates, represented by 1–2 isolates each (Table 1). Of note, genotype 4.3.1 (H58), which has become dominant elsewhere in sub-Saharan Africa and accounts for the majority of antimicrobial resistant typhoid globally, was not detected in the Nigerian studies.

### Antimicrobial resistant *S*. Typhi in Nigeria

**Fig 2:**
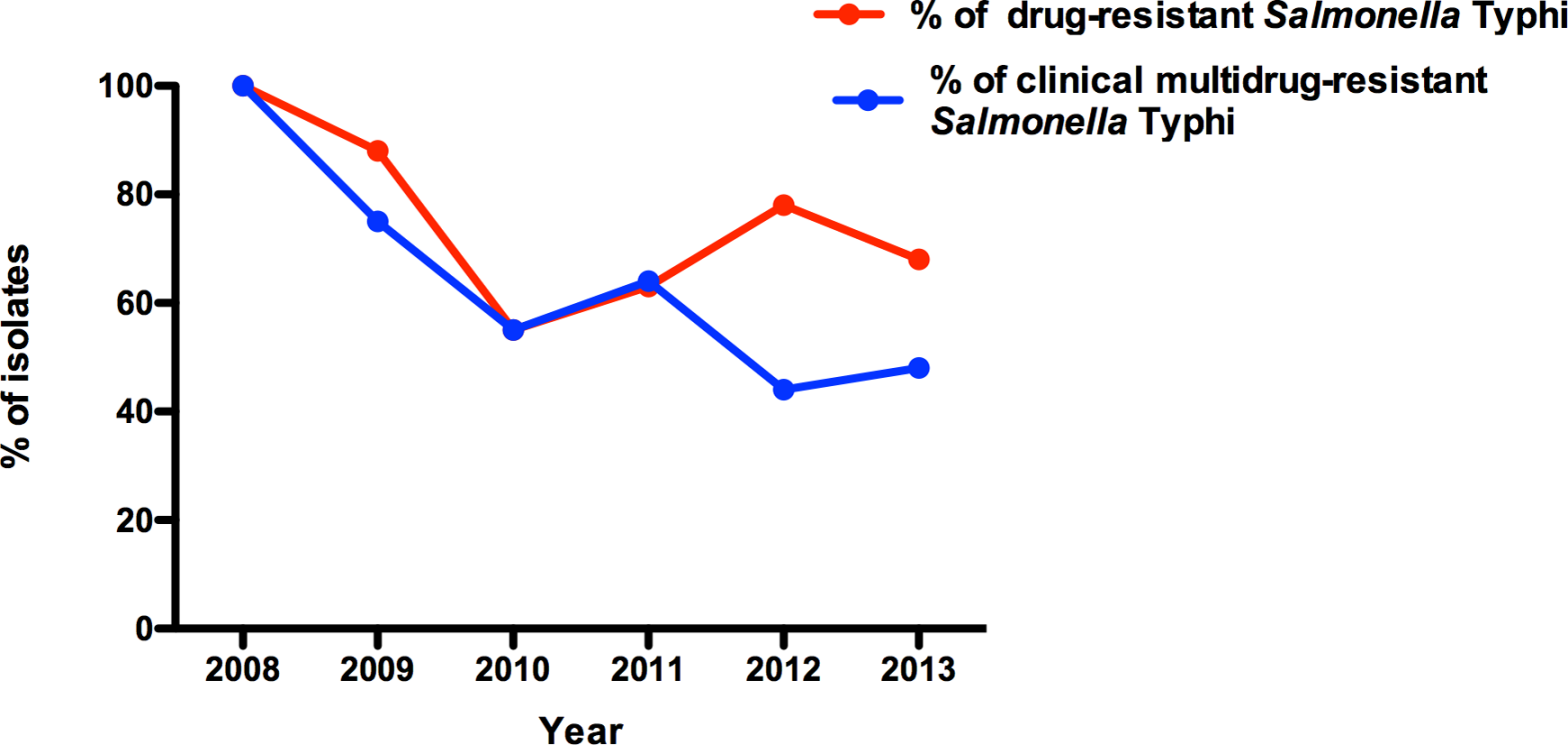
Presence of antimicrobial resistance of *S*. Typhi in the study areas. The proportion of *S*. Typhi isolates that were resistant to one or more antimicrobials (red line) and were multidrug-resistant (MDR; defined as resistance to ampicillin, chloramphenicol and trimethoprim-sulfamethoxazole, blue line) are shown. Percentages are of the total *S*. Typhi isolated per year.

Fig 2 shows the proportion of *S*. Typhi isolates that were resistant to one or more antimicrobials, and the proportion that were multidrug-resistant (MDR; defined as resistance to ampicillin, chloramphenicol and trimethoprim-sulfamethoxazole), each year from 2008–2013. The majority of isolates were MDR throughout this period (Fig 2).

**Fig 3:**
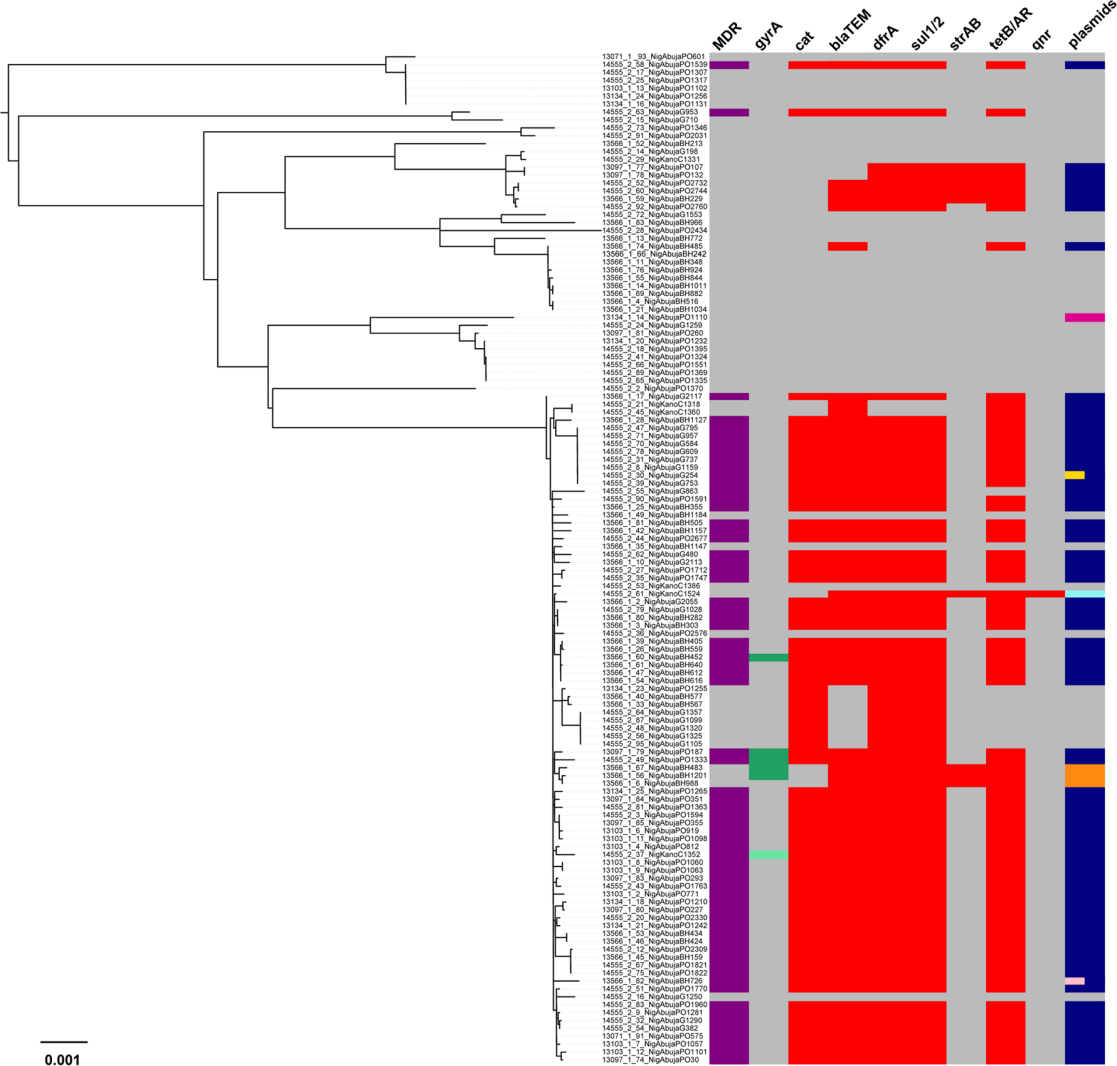
Acquired multidrug-resistance in Nigerian *S*. Typhi isolates. Maximum likelihood tree of 128 Nigerian *S*. Typhi isolates from 2,541 SNPs is shown on the left. On the right is a heatmap which shows, for each isolate, its multidrug-resistant (MDR) status (purple), the presence of *gyrA* mutations (dark green S83Y; light green S83F), resistance genes *cat*, *blaTEM*, *dfrA*, *sul1/2*, *strAB*, *tetB/AR*, *qnr* (red) and plasmids, including IncHI1 (dark blue), Kpn3 (light blue), IncY (orange), IncQ1 (light pink), IncFIIs (yellow) and Col(RNAI) (magenta). Different colored bars within the plasmid column show isolates that harbor multiple plasmids with each bar representing a plasmid type. The absence of a genotype or plasmid was displayed in grey. Branch lengths are indicative of the estimated substitution rate per variable site.

Fig 3 and Table 2 show the distribution of antimicrobial resistance determinants in the Nigerian isolates. Most of the 3.1.1 (H56) isolates carried genes encoding resistance to ampicillin, chloramphenicol, tetracycline and sulfamethoxazole (*bla*_TEM-1_, *catA1*, *tetB*, *dfrA15*, *sul1*). These were located on an IncHI1 plasmid, similar to that commonly found in MDR *S*. Typhi 4.3.1 (H58). The same profile was identified in a single isolate of 0.0.3, indicative of local plasmid transfer between the co-circulating genotypes. Genotype 2.3.1 isolates were found to carry IncHI1 plasmids encoding these resistance genes, as well as resistance determinants *sul2* and *strAB*. An IncHI1 plasmid carrying *bla*_TEM_ and *tetB* was also identified in one 2.2.0 isolate. Interestingly, nine genotype 3.1.1 isolates lacked the IncHI1 plasmid. However, four of these carried plasmids of other incompatibility groups. Three isolates (3135STDY5861338; 3135STDY5861351; 3135STDY5861282) harbored a novel IncY plasmid (*bla*_TEM-198_, *catA1*, *tetB*, *dfrA14*, *sul1*) and one (3135STDY5861242) harbored a plasmid-related to the Kpn3 plasmid (*bla*_TEM-198_, *tetAR*, *dfrA14*, *sul1*, *sul2*, *strAB* and also *qnr-S*, which mediates fluoroquinolone resistance). Thus, plasmid-mediated MDR is common in Nigerian *S*. Typhi from the regions under study.

**Table 2:** Summary of drug resistance of Nigerian *S*. Typhi.

Laboratory name	Subclade	Plasmids	Resistance genes		*gyrA* mutations
3135STDY5861196	0.0.1	-	-		-
3135STDY5861244	0.0.1	-	cat, dfrA, sul1, bla-TEM, tetB		-
PO_601	0.0.3	-	-		-
PO_1102	0.0.3	-	-		-
PO_1131	0.0.3	-	-		-
3135STDY5861198	0.0.3	-	-		-
3135STDY5861206	0.0.3	-	-		-
3135STDY5861239	0.0.3	IncHI1	cat, dfrA, sul1, bla-TEM, tetB		-
PO_1256	0.0.3	-	-		-
3135STDY5861254	2.1.0	-	-		-
3135STDY5861272	2.1.0	-	-		-
3135STDY5861209	2.2.0	-	-		-
3135STDY5861359	2.2.0	IncHI1	bla-TEM, tetB		-
3135STDY5861290	2.2.0	-	-		-
3135STDY5861298	2.2.0	-	-		-
3135STDY5861350	2.2.0	-	-		-
3135STDY5861287	2.2.0	-	-		-
3135STDY5861280	2.2.0	-	-		-
3135STDY5861289	2.2.0	-	-		-
3135STDY5861337	2.2.0	-	-		-
3135STDY5861353	2.2.0	-	-		-
3135STDY5861361	2.2.0	-	-		-
3135STDY5861369	2.2.0	-	-		-
3135STDY5861253	2.2.0	-	-		-
PO_132	2.3.1	IncHI1	dfrA, sul1, tetB, strA, strB, sul2, aad		-
PO_107	2.3.1	IncHI1	dfrA, tetB, strA, strB, sul2, aad		-
3135STDY5861233	2.3.1	IncHI1	dfrA, sul1, bla-TEM, tetB, strA, strB, sul2, aad		-
3135STDY5861241	2.3.1	IncHI1	dfrA, sul1, bla-TEM, tetB, strA, strB, sul2, aad		-
3135STDY5861273	2.3.1	IncHI1	dfrA, sul1, bla-TEM, tetB, aad		-
3135STDY5861210	2.3.1	-	-		-
3135STDY5861195	2.3.1	-	-		-
3135STDY5861342	2.3.1	IncHI1	dfrA, sul1, bla-TEM, tetB, strA, strB, sul2, aad		-
3135STDY5861334	2.3.2	-	-		-
3135STDY5861294	3.1.0	IncHI1	cat, dfrA, sul1, bla-TEM, tetB		-
PO_30	3.1.1	IncHI1	cat, dfrA, sul1, bla-TEM, tetB		-
PO_1057	3.1.1	IncHI1	cat, dfrA, sul1, bla-TEM, tetB		-
PO_1060	3.1.1	IncHI1	cat, dfrA, sul1, bla-TEM, tetB		-
PO_1063	3.1.1	IncHI1	cat, dfrA, sul1, bla-TEM, tetB		-
PO_187	3.1.1	IncHI1	cat, dfrA, sul1, bla-TEM, tetB		S83Y
PO_227	3.1.1	IncHI1	cat, dfrA, sul1, bla-TEM, tetB		-
PO_293	3.1.1	IncHI1	cat, dfrA, sul1, bla-TEM, tetB		-
PO_351	3.1.1	IncHI1	cat, dfrA, sul1, bla-TEM, tetB		-
PO_355	3.1.1	IncHI1	cat, dfrA, sul1, bla-TEM, tetB		-
PO_771	3.1.1	IncHI1	cat, dfrA, sul1, bla-TEM, tetB		-
PO_812	3.1.1	IncHI1	cat, dfrA, sul1, bla-TEM, tetB		-
PO_919	3.1.1	IncHI1	cat, dfrA, sul1, bla-TEM, tetB		-
PO_575	3.1.1	IncHI1	cat, dfrA, sul1, bla-TEM, tetB		-
PO_1255	3.1.1	-	cat, dfrA, sul1		-
PO_1098	3.1.1	IncHI1	cat, dfrA, sul1, bla-TEM, tetB		-
PO_1101	3.1.1	IncHI1	cat, dfrA, sul1, bla-TEM, tetB		-
PO_1210	3.1.1	IncHI1	cat, dfrA, sul1, bla-TEM, tetB		-
PO_1242	3.1.1	IncHI1	cat, dfrA, sul1, bla-TEM, tetB		-
PO_1265	3.1.1	IncHI1	cat, dfrA, sul1, bla-TEM, tetB		-
3135STDY5861190	3.1.1	IncHI1	cat, dfrA, sul1, bla-TEM, tetB		-
3135STDY5861230	3.1.1	IncHI1	cat, dfrA, sul1, bla-TEM, tetB		S83Y
3135STDY5861262	3.1.1	IncHI1	cat, dfrA, sul1, bla-TEM, tetB		-
3135STDY5861271	3.1.1	IncHI1	cat, dfrA, sul1, bla-TEM, tetB		-
3135STDY5861184	3.1.1	IncHI1	cat, dfrA, sul1, bla-TEM, tetB		-
3135STDY5861208	3.1.1	IncHI1	cat, dfrA, sul1, bla-TEM, tetB		-
3135STDY5861216	3.1.1	IncHI1	cat, dfrA, sul1, bla-TEM, tetB		-
3135STDY5861224	3.1.1	IncHI1	cat, dfrA, sul1, bla-TEM, tetB		-
3135STDY5861232	3.1.1	IncHI1	cat, dfrA, sul1, bla-TEM, tetB		-
3135STDY5861248	3.1.1	IncHI1	cat, dfrA, sul1, bla-TEM, tetB		-
3135STDY5861256	3.1.1	IncHI1	cat, dfrA, sul1, bla-TEM, tetB		-
3135STDY5861264	3.1.1	IncHI1	cat, dfrA, sul1, bla-TEM, tetB		-
3135STDY5861193	3.1.1	IncHI1	cat, dfrA, sul1, bla-TEM, tetB		-
3135STDY5861201	3.1.1	IncHI1	cat, dfrA, sul1, bla-TEM, tetB		-
3135STDY5861217	3.1.1	-	-		-
3135STDY5861225	3.1.1	IncHI1	cat, dfrA, sul1, bla-TEM, tetB		-
3135STDY5861226	3.1.1	IncHI1	bla-TEM, tetB		-
3135STDY5861202	3.1.1	IncHI1	bla-TEM, tetB		-
3135STDY5861234	3.1.1	-	-		-
3135STDY5861242	3.1.1	Kpn3	bla-TEM, strA, strB, sul1, sul2, dfrA, tetA, tetR, qnrS		-
3135STDY5861218	3.1.1	IncHI1	cat, dfrA, sul1, bla-TEM, tetB		S83F
3135STDY5861211	3.1.1	IncHI1, IncFIIs	cat, dfrA, sul1, bla-TEM, tetB		-
3135STDY5861235	3.1.1	IncHI1	cat, dfrA, sul1, bla-TEM, tetB		-
3135STDY5861243	3.1.1	IncHI1	cat, dfrA, sul1, bla-TEM, tetB		-
3135STDY5861312	3.1.1	-	cat, dfrA, sul1		-
3135STDY5861320	3.1.1	-	cat, dfrA, sul1		-
3135STDY5861268	3.1.1	-	cat, dfrA, sul1		-
3135STDY5861276	3.1.1	-	cat, dfrA, sul1		-
3135STDY5861229	3.1.1	-	cat, dfrA, sul1		-
3135STDY5861237	3.1.1	-	cat, dfrA, sul1		-
3135STDY5861245	3.1.1	-	cat, dfrA, sul1		-
3135STDY5861314	3.1.1	-	-		-
3135STDY5861330	3.1.1	-	-		-
3135STDY5861197	3.1.1	-	-		-
3135STDY5861338	3.1.1	IncY	bla-TEM, strA, strB, sul1, dfrA, tetA, tetR		S83Y
3135STDY5861351	3.1.1	IncY	bla-TEM, strA, strB, sul1, dfrA, tetA, tetR		S83Y
3135STDY5861282	3.1.1	IncY	bla-TEM, strA, strB, sul1, dfrA, tetA, tetR		S83Y
3135STDY5861306	3.1.1	IncHI1	cat, dfrA, sul1, bla-TEM, tetB		-
3135STDY5861322	3.1.1	IncHI1	cat, dfrA, sul1, bla-TEM, tetB		-
3135STDY5861326	3.1.1	IncHI1	cat, dfrA, sul1, bla-TEM, tetB		-
3135STDY5861366	3.1.1	IncHI1	cat, dfrA, sul1, bla-TEM, tetB		-
3135STDY5861279	3.1.1	IncHI1	cat, dfrA, sul1, bla-TEM, tetB		-
3135STDY5861303	3.1.1	IncHI1	cat, dfrA, sul1, bla-TEM, tetB		-
3135STDY5861319	3.1.1	IncHI1	cat, dfrA, sul1, bla-TEM, tetB		-
3135STDY5861327	3.1.1	IncHI1	cat, dfrA, sul1, bla-TEM, tetB		-
3135STDY5861335	3.1.1	IncHI1	cat, dfrA, sul1, bla-TEM, tetB		-
3135STDY5861343	3.1.1	IncHI1	cat, dfrA, sul1, bla-TEM, tetB		-
3135STDY5861367	3.1.1	IncHI1	cat, dfrA, sul1, bla-TEM, tetB		-
3135STDY5861304	3.1.1	IncHI1	cat, dfrA, sul1, bla-TEM, tetB		-
3135STDY5861328	3.1.1	IncHI1	cat, dfrA, sul1, bla-TEM, tetB		-
3135STDY5861336	3.1.1	IncHI1	cat, dfrA, sul1, bla-TEM, tetB		-
3135STDY5861344	3.1.1	IncHI1	cat, dfrA, sul1, bla-TEM, tetB		-
3135STDY5861260	3.1.1	IncHI1	cat, dfrA, sul1, bla-TEM, tetB		-
3135STDY5861189	3.1.1	IncHI1	cat, dfrA, sul1, bla-TEM, tetB		-
3135STDY5861213	3.1.1	IncHI1	cat, dfrA, sul1, bla-TEM, tetB		-
3135STDY5861278	3.1.1	IncHI1	cat, dfrA, sul1, bla-TEM, tetB		-
3135STDY5861286	3.1.1	IncHI1	5	cat, dfrA, sul1, bla-TEM, tetB	-
3135STDY5861251	3.1.1	IncHI1	5	cat, dfrA, sul1, bla-TEM, tetB	-
3135STDY5861259	3.1.1	IncHI1	5	cat, dfrA, sul1, bla-TEM, tetB	-
3135STDY5861212	3.1.1	IncHI1	5	cat, dfrA, sul1, bla-TEM, tetB	-
3135STDY5861220	3.1.1	IncHI1	5	cat, dfrA, sul1, bla-TEM, tetB	-
3135STDY5861228	3.1.1	IncHI1	5	cat, dfrA, sul1, bla-TEM, tetB	-
3135STDY5861252	3.1.1	IncHI1	5	cat, dfrA, sul1, bla-TEM, tetB	-
3135STDY5861236	3.1.1	IncHI1	4	cat, dfrA, sul1, bla-TEM,	-
3135STDY5861368	3.1.1	IncHI1, IncQ1	5	cat, dfrA, sul1, bla-TEM, tetB	-
3135STDY5861183	3.3.0	-	0	-	-
PO_260	4.1.0	-	0	-	-
PO_1232	4.1.0	-	0	-	-
3135STDY5861222	4.1.0	-	0	-	-
3135STDY5861246	4.1.0	-	0	-	-
3135STDY5861270	4.1.0	-	0	-	-
3135STDY5861199	4.1.0	-	0	-	-
3135STDY5861247	4.1.0	-	0	-	-
3135STDY5861205	4.1.0	-	0	-	-
PO_1110	4.1.1	Col(RNAI)	0	-	-

We identified only six *S*. Typhi isolates with quinolone resistance-associated mutations in *gyrA* (one with S83F; five with S83Y). The affected isolates were all of the dominant genotype 3.1.1, including the three that carried IncY plasmids and three that carried IncHI1 plasmids. No other polymorphisms were detected in the quinolone resistance determining regions of the *gyrA* or *parC* genes of Nigerian *S*. Typhi isolates.

## Discussion

Here, *S*. Typhi is shown to be a common cause of bacteremia and fever among children living in two geographically distinct regions of Nigeria. Studies on typhoid within Nigeria have been relatively rare, even though it is a country with a large population and extensive urbanization. Indeed, *S*. Typhi is the most common bacterial cause of bloodstream infections. Phylogenetic analysis identified distinct clusters of *S*. Typhi, with isolates of genotype 3.1.1 representing 66% of all isolates. Other common genotypes included 2.2.0 and 2.3.1, which have been previously reported in Africa, and genotypes 4.1.0 and 0.0.3, which were previously reported in Asia. The presence of multiple genotypes in these comparatively small regions suggests typhoid has been established for some time and that different waves of disease have entered the regions at different times. It is also interesting that the different clades of Nigerian isolates distributed across the phylogeny frequently map adjacent to other *S*. Typhi isolates from other African countries. For example, genotype 3.1.1 maps adjacent to *S*. Typhi isolates from both west and north Africa, with the Nigerian isolates located on a more recent phylogenetic branch. Similarly, genotypes 2.2.0 and 2.3.1 also map close to other African isolates. This general distribution indicates substantial exchange of *S*. Typhi between Nigeria and other parts of Africa. However, the phylogenetic analysis was limited to two sites within Nigeria, with only six *S*. Typhi isolates included in the analysis from Kano, over a five- year period, resulting in a selection bias towards strains from a single study site in Nigeria (Abuja). Therefore, a more comprehensive analysis involving a larger number of strains from multiple regions across Nigeria and surrounding countries over a wider time span would be required to further investigate transmission within the region.

It is notable that none of the Nigerian isolates were of the genotype 4.3.1 (H58), which is now expanding across many other regions with endemic typhoid and is associated with a MDR phenotype. This suggests that the recent expansion of H58 *S*. Typhi, estimated to date from the mid-1980s, has not yet reached Nigeria, unlike other African countries including Kenya, Tanzania, Malawi and South Africa. The absence of H58 isolates in the sampled area of Nigeria is an important finding. It has been postulated that H58 *S*. Typhi originally emerged in Asia, but subsequently entered Africa on a number of distinct occasions where they have gone on to cause large typhoid outbreaks [12]. Thus, it is likely that H58 *S*. Typhi will reach Nigeria in the future, potentially changing the epidemiology of the disease in the region and molecular surveillance could be used to monitor for this.

Nevertheless, MDR *S*. Typhi are common in the regions of study despite the absence of H58 microorganisms. This is an important observation, as the MDR phenotype in other regions of the world has been driven by the spread of MDR *S*. Typhi H58. Many of the Nigerian *S*. Typhi, including those of genotype 3.1.1, harbored IncHI1 plasmids that have been previously associated with *S*. Typhi of other genotypes, particularly H58 [12, 39]. This is consistent with a genetic compatibility between *S*. Typhi and such plasmids. Interestingly, genetic analysis indicates that an IncHI1 plasmid recently transferred between 3.1.1 and 0.0.3 Typhi within the study region. However, several other plasmids of distinct incompatibility types were also detected within the sampled *S*. Typhi and it will be interesting to see if any of these are common elsewhere in Nigeria or whether they solely persist within these study sites.

Mutations associated with resistance to quinolones were relatively rare within the sample set. This could be because fluoroquinolones are not commonly used to treat typhoid in these regions, or alternatively, it may be that such mutations have not become fixed in these non-H58 isolates. Further studies on the use of fluoroquinolones are warranted.

In conclusion, it is clear that typhoid associated with MDR *S*. Typhi is common in these parts of Nigeria and that the MDR phenotype is evolving independently of haplotype H58, which has emerged elsewhere in the world where typhoid is endemic.

### Members of International Typhoid Consortium

Vanessa K. Wong^1,2^, Stephen Baker^3,4,5^, Derek Pickard^1^, Julian Parkhill^1^, Andrew J Page^1^, Nicholas A. Feasey^6^ Robert A. Kingsley^1,7^, Nicholas R. Thomson^1,5^, Jacqueline A. Keane^1^, François-Xavier Weill^8^, Simon Le Hello^8^, Jane Hawkey^9,10,11^, David J. Edwards^9,11^, Zoe A. Dyson^9,11^, Simon R. Harris^1^, Amy K. Cain^1^, James Hadfield^1^, Peter J. Hart^12,13^, Nga Tran Vu Thieu^3^, Elizabeth J. Klemm^1^, Robert F. Breiman^14,15,16^, Conall H. Watson^17^, Samuel Kariuki^1,14^, Melita A. Gordon^18,19^, Robert S. Heyderman^20,19,^ Chinyere Okoro^1,2^, Jan Jacobs^21,22^, Octavie Lunguya^23,24^, W. John Edmunds^17^, Chisomo Msefula^19,25^, Jose A. Chabalgoity^26^, Mike Kama^27^, Kylie Jenkins^28^, Shanta Dutta^29^, Florian Marks^30^, Josefina Campos^31^, Corinne Thompson^3,4^, Stephen Obaro^32,33,34^, Calman A. MacLennan^1,12,35^, Christiane Dolecek^3,4^, Karen H. Keddy^36^, Anthony M. Smith^36^, Christopher M. Parry^37,38^, Abhilasha Karkey^39^, E. Kim Mulholland^5,40^, James I. Campbell^3,4^, Sabina Dongol^39^, Buddha Basnyat^39^, Amit Arjyal^39^, Muriel Dufour^41^, Don Bandaranayake^42^, Take N. Toleafoa^43^, Shalini Pravin Singh^44^, Mochammad Hatta^45^, Robert S. Onsare^14^, Lupeoletalalelei Isaia^46^, Guy Thwaites^3,4^, Paul Turner^4,47,48^, Sona Soeng^48^, John A. Crump^49^, Elizabeth De Pinna^50^, Satheesh Nair^50^, Eric J Nille^51^, Duy Pham Thanh^3^, Mary Valcanis^52^, Joan Powling^52^, Karolina Dimovski^52^, Geoff Hogg^52^, Thomas R. Connor^53^, Jayshree Dave^54^, Niamh Murphy^54^, Richard Holliman^54^, Armine Sefton^55^, Michael Millar^55^, Jeremy Farrar^3,4^, Alison E. Mather^56^, Ben Amos^57^, Grace Olanipekun^58^, Huda Munir^59^, Roxanne Alter^60^, Paul D. Fey^60^, Kathryn E Holt^9,11^ and Gordon Dougan^1^

1. The Wellcome Trust Sanger Institute, Hinxton, Cambridge, United Kingdom
2. Addenbrooke’s Hospital, Cambridge University Hospitals NHS Foundation Trust, Cambridge Biomedical Campus, Hills Road, Cambridge, United Kingdom
3. The Hospital for Tropical Diseases, Wellcome Trust Major Overseas Programme, Oxford University Clinical Research Unit, Ho Chi Minh City, Vietnam
4. Centre for Tropical Medicine and Global Health, Nuffield Department of Clinical Medicine, Oxford University, Oxford, UK
5. Department of Infectious and Tropical Diseases, London School of Hygiene and Tropical Medicine, London, United Kingdom
6. Liverpool School of Tropical Medicine, Pembroke Place, Liverpool, United Kingdom
7. Institute of Food Research, Norwich Research Park, Colney, Norwich, United Kingdom
8. Institut Pasteur, Unité des Bactéries Pathogènes Entériques, Paris, France
9. Department of Biochemistry and Molecular Biology, Bio21 Molecular Science and Biotechnology Institute, University of Melbourne, Parkville, Victoria, Australia
10. Faculty of Veterinary and Agricultural Sciences, University of Melbourne, Parkville, Victoria, Australia
11. Centre for Systems Genomics, University of Melbourne, Parkville, Victoria, Australia
12. Institute of Biomedical Research, School of Immunity and Infection, College of Medicine and Dental Sciences, University of Birmingham, Birmingham, United Kingdom
13. St George’s University of London, London, United Kingdom
14. Kenya Medical Research Institute (KEMRI), Nairobi, Kenya
15. Centers for Disease Control and Prevention, Atlanta, Georgia, United States of America
16. Emory Global Health Institute, Atlanta, Georgia, United States of America
17. Centre for the Mathematical Modelling of Infectious Diseases, Department of Infectious Disease Epidemiology, London School of Hygiene and Tropical Medicine, Keppel Street, London, United Kingdom
18. Institute of Infection and Global Health, University of Liverpool, United Kingdom
19. Malawi-Liverpool-Wellcome-Trust Clinical Research Programme, College of Medicine, University of Malawi, Chichiri, Blantyre, Malawi
20. Division of Infection and Immunity, University College London, London, United Kingdom
21. Department of Clinical Sciences, Institute of Tropical Medicine, Antwerp, Belgium
22. KU Leuven, University of Leuven, Department of Microbiology and Immunology, Belgium
23. National Institute for Biomedical Research, Kinshasa, Democratic Republic of the Congo
24. University Hospital of Kinshasa, Kinshasa, Democratic Republic of the Congo
25. Microbiology Department, College of Medicine, University of Malawi, Malawi
26. Departamento de Desarrollo Biotecnologico, Instituto de Higiene, Facultad de Medicina, Avda A Navarro 3051, Montevideo, Uruguay
27. Ministry of Health, Toorak, Suva, Fiji
28. Fiji Health Sector Support Program, Suva, Fiji
29. National Institute of Cholera and Enteric Diseases, Scheme XM, Beliaghata, Kolkata, India
30. International Vaccine Institute, Department of Epidemiology, Kwanak, Republic of Korea
31. Enteropathogen Division, ANLIS-Carlos G Malbran Institute, CABA, Argentina
32. Division of Pediatric Infectious Diseases, University of Nebraska Medical Center, Omaha, Nebraska, United States of America
33. University of Abuja Teaching Hospital, Gwagwalada, FCT, Nigeria
34. Bingham University, Karu, Nassarawa State, Nigeria
35. The Jenner Institute, Nuffield Department of Medicine, University of Oxford, Oxford, United Kingdom
36. Centre for Enteric Diseases, National Institute for Communicable Diseases, Division in the National Health Laboratory Service and Faculty of Health Sciences, University of the Witwatersrand, Johannesburg, South Africa
37. Department of Clinical Research, London School of Hygiene and Tropical Medicine, Keppel Street, London, United Kingdom
38. Graduate School of Tropical Medicine and Global Health, Nagasaki University, Nagasaki, Japan
39. Patan Academy of Health Sciences, Wellcome Trust Major Overseas Programme, Oxford University Clinical Research Unit, Kathmandu, Nepal
40. Murdoch Childrens Research Institute, Melbourne, Australia
41. Enteric and *Leptospira* Reference Laboratory, Institute of Environmental Science and Research Limited (ESR), New Zealand
42. National Centre for Biosecurity and Infectious Disease, Institute of Environmental Science and Research, Porirua, New Zealand
43. Samoa Ministry of Health, Apia, Samoa
44. National Influenza Center, World Health Organization, Center for Communicable Disease Control, Suva, Fiji
45. Department of Microbiology, Hasanuddin University, Makassar, Indonesia
46. National Health Services, Tupua Tamasese Meaole Hospital, Samoa
47. Mahidol-Oxford Tropical Medicine Research Unit, Faculty of Tropical Medicine, Mahidol University, Bangkok, Thailand
48. Cambodia-Oxford Medical Research Unit, Angkor Hospital for Children, Siem Reap, Cambodia
49. Centre for International Health, University of Otago, Dunedin, New Zealand
50. Salmonella Reference Service, Public Health England, Colindale, London, United Kingdom
51. Emerging Disease Surveillance and Response, Division of Pacific Technical Support, World Health Organization, Suva, Fiji
52. Microbiological Diagnostic Unit—Public Health Laboratory, Department of Microbiology and Immunology at the Peter Doherty Institute for Infection and Immunity, The University of Melbourne, Victoria, Australia
53. Cardiff University School of Biosciences, Cardiff University, Cardiff, United Kingdom
54. Public Health Laboratory London, Public Health England, London, United Kingdom
55. Division of Infection, Barts Health NHS Trust, London, United Kingdom
56. Department of Veterinary Medicine, University of Cambridge, Cambridge, United Kingdom
57. St Augustine’s Hospital, Muheza, Tanzania
58. International Foundation Against Infectious Diseases in Nigeria, Abuja, Nigeria
59. Department of Medical Microbiology, Aminu Kano Teaching Hospital, Kano, Nigeria
60. Department of Pathology and Microbiology, University of Nebraska Medical Center, Omaha, Nebraska, United States of America

## Supporting Information

S1 Fig Global distribution of African *S*. Typhi isolates analyzed in this study. A maximum likelihood tree of 1,960 *S*. Typhi isolates from 23,300 SNPs surrounded by colored rings representing the geographic origin of 502 African isolates, according to the legend. 128 Nigerian isolates are highlighted in black (122 = Abuja) and grey (6 = Kano); neighboring African countries labeled by black arrows. The genotypes of the Nigerian isolates are labeled in red with the old Roumagnac haplotypes [11] in parentheses (red * denotes untypeable Nigerian strains). The 4.3.1 (H58) subclade is indicated in red italics. Branch lengths are indicative of the estimated substitution rate per variable site.
(TIF)

S1 Table Details of the 128 Nigerian *Salmonella* Typhi isolates used in the study (see excel sheet). (XLS)

## References

[1] Crump JA, Luby SP, Mintz ED. The global burden of typhoid fever. Bull World Health Organ. 2004;82(5):346–53. Epub 2004/08/10. 15298225 PMC2622843

[2] Crump JA, Mintz ED. Global trends in typhoid and paratyphoid Fever. Clin Infect Dis. 2010;50(2):241–6. 10.1086/649541 20014951 PMC2798017

[3] Mogasale V, Maskery B, Ochiai RL, Lee JS, Mogasale VV, Ramani E, et al Burden of typhoid fever in low-income and middle-income countries: a systematic, literature-based update with risk-factor adjustment. The Lancet Global health. 2014;2(10):e570–80. 10.1016/S2214-109X(14)70301-8 . 25304633

[4] Parry CM, Hien TT, Dougan G, White NJ, Farrar JJ. Typhoid fever. N Engl J Med. 2002;347(22):1770–82. 10.1056/NEJMra020201 . 12456854

[5] Enwere G, Biney E, Cheung YB, Zaman SM, Okoko B, Oluwalana C, et al Epidemiologic and clinical characteristics of community-acquired invasive bacterial infections in children aged 2–29 months in The Gambia. Pediatr Infect Dis J. 2006;25(8):700–5. 10.1097/01.inf.0000226839.30925.a5 . 16874169

[6] Berkley JA, Lowe BS, Mwangi I, Williams T, Bauni E, Mwarumba S, et al Bacteremia among children admitted to a rural hospital in Kenya. N Engl J Med. 2005;352(1):39–47. 10.1056/NEJMoa040275 . 15635111

[7] Isendahl J, Manjuba C, Rodrigues A, Xu W, Henriques-Normark B, Giske CG, et al Prevalence of community-acquired bacteraemia in Guinea-Bissau: an observational study. BMC Infect Dis. 2014;14:3859 10.1186/s12879-014-0715-9 25526763 PMC4297428

[8] Arulogun OS, Adeniyi JD, Asa S, Adegbenro CA. Why actions for early treatment of febrile illnesses in children are delayed by caregivers. Int Q Community Health Educ. 2011;32(3):219–31. 10.2190/IQ.32.3.e . 23353563

[9] Obaro SK, Hassan-Hanga F, Olateju EK, Umoru D, Lawson L, Olanipekun G, et al Salmonella Bacteremia Among Children in Central and Northwest Nigeria, 2008–2015. Clin Infect Dis. 2015;61 Suppl 4:S325–31. 10.1093/cid/civ745 26449948 PMC4596937

[10] Holt KE, Parkhill J, Mazzoni CJ, Roumagnac P, Weill FX, Goodhead I, et al High-throughput sequencing provides insights into genome variation and evolution in Salmonella Typhi. Nat Genet. 2008;40(8):987–93. Epub 2008/07/29. ng.195 [pii] 10.1038/ng.195 18660809 PMC2652037

[11] Roumagnac P, Weill FX, Dolecek C, Baker S, Brisse S, Chinh NT, et al Evolutionary history of Salmonella typhi. Science. 2006;314(5803):1301–4. Epub 2006/11/25. 314/5803/1301 [pii] 10.1126/science.1134933 17124322 PMC2652035

[12] Wong VK, Baker S, Pickard DJ, Parkhill J, Page AJ, Feasey NA, et al Phylogeographical analysis of the dominant multidrug-resistant H58 clade of Salmonella Typhi identifies inter- and intracontinental transmission events. Nat Genet. 2015;47(6):632–9. 10.1038/ng.3281 . 25961941 PMC4921243

[13] The World Bank 2015. http://data.worldbank.org/country/nigeria. Accessed 18th November 2015.

[14] Abuja, the Beautiful Capital city (21 January 2011).Available at: http://9ja-land.blogstop.com/2010/05/abuja-beautiful-capital-city.html. Accessed 18 November 2015.

[15] Obaro S, Lawson L, Essen U, Ibrahim K, Brooks K, Otuneye A, et al Community acquired bacteremia in young children from central Nigeria—a pilot study. BMC Infect Dis. 2011;11:137 10.1186/1471-2334-11-137 21595963 PMC3111365

[16] Federal Republic of Nigeria NAftCoA, Global AIDS Response Country Progress Report 2014. Available at: http://www.unaids.org/sites/default/files/country/documents/NGA_narrative_report_2014.pdf. Accessed 11 November 2015.

[17] Olayemi IK, Ande AT, Ayanwale AV, Mohammed AZ, Bello IM, Idris B, et al Seasonal trends in epidemiological and entomological profiles of malaria transmission in North Central Nigeria. Pak J Biol Sci. 2011;14(4):293–9. . 21870632 10.3923/pjbs.2011.293.299

[18] Federal Republic of Nigeria NBoSpc, Kano state statisical table: 2010. Available at: http://www.nigriast.gov.ng. Accessed 11 November 2015.

[19] Harris PA, Taylor R, Thielke R, Payne J, Gonzalez N, Conde JG. Research electronic data capture (REDCap)—a metadata-driven methodology and workflow process for providing translational research informatics support. J Biomed Inform. 2009;42(2):377–81. 10.1016/j.jbi.2008.08.010 18929686 PMC2700030

[20] IBM Corp. Released 2013. IBM SPSS Statistics for Macintosh VA, NY: IBM Corp.

[21] Clinical and Laboratory Standards Institute. Performance Standards for Antimicrobial Susceptibility Testing: Twentieth Informational Supplement M100-S20. Wayne, PA, USA: CLSI; 2014.

[22] CDC standard protocol: molecular determination of serotype in Salmonella version 2.0 AawcucgciAN.

[23] Herrera-Leon S, McQuiston JR, Usera MA, Fields PI, Garaizar J, Echeita MA. Multiplex PCR for distinguishing the most common phase-1 flagellar antigens of Salmonella spp. J Clin Microbiol. 2004;42(6):2581–6. 10.1128/JCM.42.6.2581–2586.2004 15184437 PMC427890

[24] Fitzgerald C, Collins M, van Duyne S, Mikoleit M, Brown T, Fields P. Multiplex, bead-based suspension array for molecular determination of common Salmonella serogroups. J Clin Microbiol. 2007;45(10):3323–34. 10.1128/JCM.00025-07 17634307 PMC2045348

[25] Croucher NJ, Harris SR, Fraser C, Quail MA, Burton J, van der Linden M, et al Rapid pneumococcal evolution in response to clinical interventions. Science. 2011;331(6016):430–4. Epub 2011/01/29. 10.1126/science.1198545 21273480 PMC3648787

[26] Parkhill J, Dougan G, James KD, Thomson NR, Pickard D, Wain J, et al Complete genome sequence of a multiple drug resistant Salmonella enterica serovar Typhi CT18. Nature. 2001;413(6858):848–52. Epub 2001/10/26. 10.1038/35101607 . 11677608

[27] Li H, Handsaker B, Wysoker A, Fennell T, Ruan J, Homer N, et al The Sequence Alignment/Map format and SAMtools. Bioinformatics. 2009;25(16):2078–9. Epub 2009/06/10. 10.1093/bioinformatics/btp352 19505943 PMC2723002

[28] Croucher NJ, Page AJ, Connor TR, Delaney AJ, Keane JA, Bentley SD, et al Rapid phylogenetic analysis of large samples of recombinant bacterial whole genome sequences using Gubbins. Nucleic Acids Res. 2015;43(3):e15 10.1093/nar/gku1196 25414349 PMC4330336

[29] Stamatakis A. RAxML-VI-HPC: maximum likelihood-based phylogenetic analyses with thousands of taxa and mixed models. Bioinformatics. 2006;22(21):2688–90. Epub 2006/08/25. 10.1093/bioinformatics/btl446 . 16928733

[30] Letunic I, Bork P. Interactive Tree Of Life (iTOL): an online tool for phylogenetic tree display and annotation. Bioinformatics. 2007;23(1):127–8. 10.1093/bioinformatics/btl529 . 17050570

[31] Letunic I, Bork P. Interactive Tree Of Life v2: online annotation and display of phylogenetic trees made easy. Nucleic Acids Res. 2011;39(Web Server issue):W475–8. 10.1093/nar/gkr201 21470960 PMC3125724

[32] Inouye MD, H. Raven LA. Schultz M.B. Pope B.J. Tomita T. Zobel J. and Holt K. E. SRST2: Rapid genomic surveillance for public health and hospital microbiology labs. Genome Medicine. 2014;6(90). 10.1186/s13073-014-0090-6 PMC4237778 25422674

[33] Gupta SK, Padmanabhan BR, Diene SM, Lopez-Rojas R, Kempf M, Landraud L, et al ARG-ANNOT, a new bioinformatic tool to discover antibiotic resistance genes in bacterial genomes. Antimicrob Agents Chemother. 2014;58(1):212–20. 10.1128/AAC.01310-13 24145532 PMC3910750

[34] Carattoli A, Zankari E, Garcia-Fernandez A, Voldby Larsen M, Lund O, Villa L, et al In silico detection and typing of plasmids using PlasmidFinder and plasmid multilocus sequence typing. Antimicrob Agents Chemother. 2014;58(7):3895–903. 10.1128/AAC.02412-14 24777092 PMC4068535

[35] Eaves DJ, Randall L, Gray DT, Buckley A, Woodward MJ, White AP, et al Prevalence of mutations within the quinolone resistance-determining region of gyrA, gyrB, parC, and parE and association with antibiotic resistance in quinolone-resistant Salmonella enterica. Antimicrob Agents Chemother. 2004;48(10):4012–5. 10.1128/AAC.48.10.4012–4015.2004 15388468 PMC521866

[36] Baucheron S, Chaslus-Dancla E, Cloeckaert A, Chiu CH, Butaye P. High-level resistance to fluoroquinolones linked to mutations in gyrA, parC, and parE in Salmonella enterica serovar Schwarzengrund isolates from humans in Taiwan. Antimicrob Agents Chemother. 2005;49(2):862–3. 10.1128/AAC.49.2.862–863.2005 15673791 PMC547372

[37] Hooper DC. Quinolone mode of action—new aspects. Drugs. 1993;45 Suppl 3:8–14. . 7689456 10.2165/00003495-199300453-00004

[38] Song Y, Roumagnac P, Weill FX, Wain J, Dolecek C, Mazzoni CJ, et al A multiplex single nucleotide polymorphism typing assay for detecting mutations that result in decreased fluoroquinolone susceptibility in Salmonella enterica serovars Typhi and Paratyphi A. J Antimicrob Chemother. 2010;65(8):1631–41. 10.1093/jac/dkq175 20511368 PMC2904664

[39] Holt KE, Phan MD, Baker S, Duy PT, Nga TV, Nair S, et al Emergence of a globally dominant IncHI1 plasmid type associated with multiple drug resistant typhoid. PLoS neglected tropical diseases. 2011;5(7):e1245 10.1371/journal.pntd.0001245 21811646 PMC3139670

