# Supplementary figures and images for: Molecular Surveillance Identifies Multiple Transmissions of Typhoid in West Africa
Source: PLoS Negl Trop Dis. 2016 Sep 22;10(9):e0004781. doi: 10.1371/journal.pntd.0004781 (PMC5033494; doi:10.1371/journal.pntd.0004781)

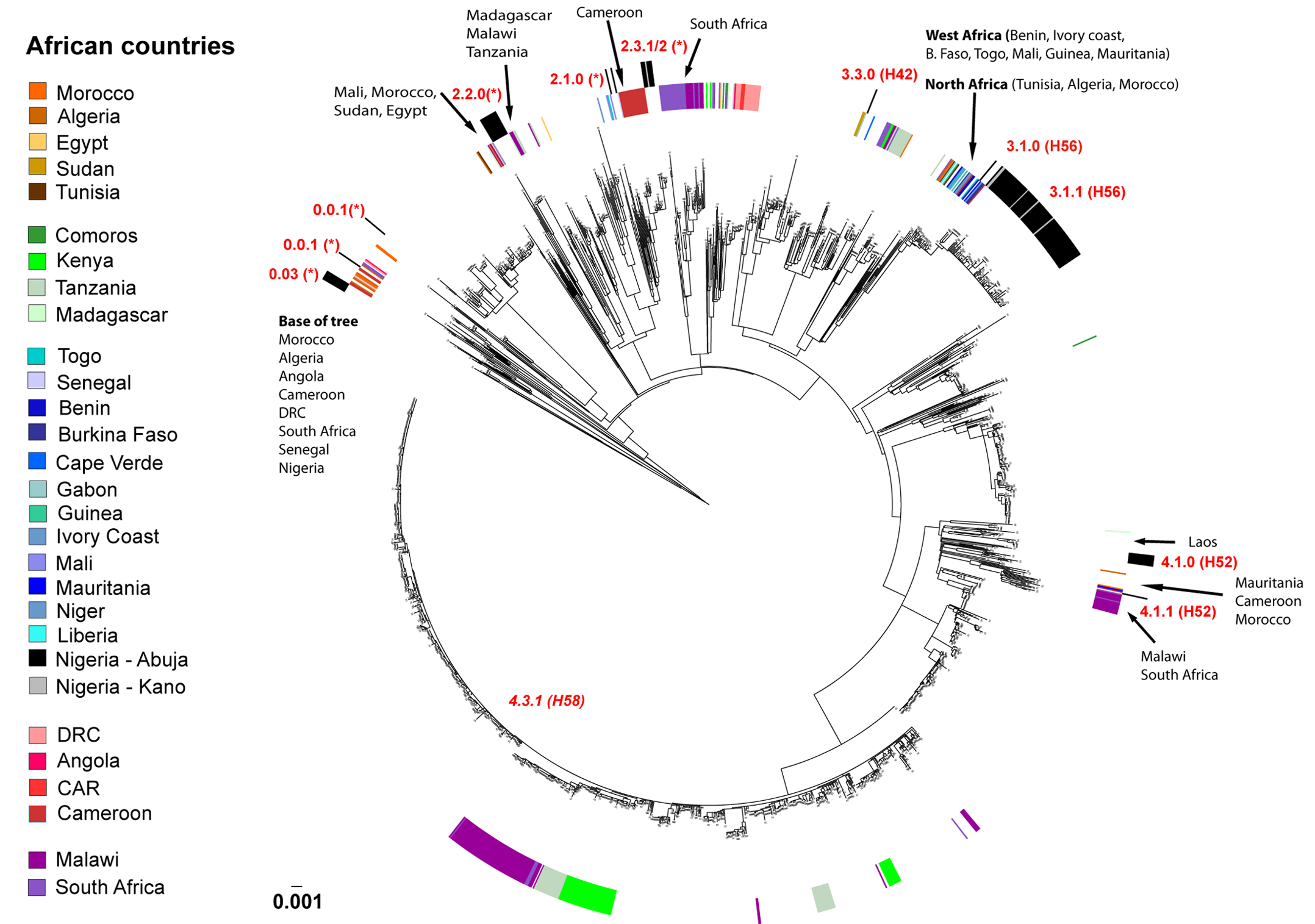

Supplement: S1 Fig — A maximum likelihood tree of 1,960 S. Typhi isolates from 23,300 SNPs surrounded by colored rings representing the geographic origin of 502 African isolates, according to the legend. 128 Nigerian isolates are highlighted in black (122 = Abuja) and grey (6 = Kano); neighboring African countries labeled by black arrows. The genotypes of the Nigerian isolates are labeled in red with the old Roumagnac haplotypes [11] in parentheses (red * denotes untypeable Nigerian strains). The 4.3.1 (H58) subclade is indicated in red italics. Branch lengths are indicative of the estimated substitution rate per variable site. (TIF) [file pntd.0004781.s001.tif]
